# Effect of pan-retinal laser photocoagulation on intravitreal vascular endothelial growth factor concentration in proliferative diabetic retinopathy

**DOI:** 10.22336/rjo.2022.49

**Published:** 2022

**Authors:** Faruk Nišić, Aida Pidro, Orhan Lepara, Almir Fajkić, Ajla Pidro Mioković, Enra Suljić, Aida Nišić, Igor Kovačević

**Affiliations:** * Clinic for Eye Disease, Clinical Centre University of Sarajevo, Sarajevo, Bosnia and Herzegovina; ** Ophthalmology Department, “Prim. Dr. Abdulah Nakaš” General Hospital, Sarajevo, Bosnia and Herzegovina; *** Department of Human Physiology, School of Medicine, University of Sarajevo, Sarajevo, Bosnia and Herzegovina; **** Department of Pathophysiology, Faculty of Medicine, University of Sarajevo, Sarajevo, Bosnia and Herzegovina; ***** Policlinic Vukas, Zagreb, Coratia; ****** Department for Science, Teaching and Clinical Trials, Clinical Centre University of Sarajevo, Sarajevo, Bosnia and Herzegovina; ******* Specialty Consultative Health Care of PI Health Centre of Sarajevo Canton, Sarajevo, Bosnia and Herzegovina; ******** Clinic for Eye disease, Clinical Centre University of Serbia, Belgrade, Serbia

**Keywords:** laser photocoagulation, proliferative diabetic retinopathy, VEGF concentration

## Abstract

**Objective:** This study aimed to determine the intravitreal concentration of VEGF in eyes with PDR and to evaluate the effects of previous PRP on its level.

**Methods:** It was a cross-sectional study performed at the Clinical Centre University. It included 90 eyes surgically treated with PPV, divided into three groups, group A - patients with PDR with previous PRP, group B - patients with PDR without previous PRP, and group C - PPV performed due to the indication unrelated to diabetes. A vitreous sample was obtained during PPV, and the VEGF concentration was determined using an Enzyme-linked immunosorbent assay test (ELISA). Shapiro-Wilk, nonparametric tests Kruskal-Wallis, Mann-Whithney U test, ANOVA and Spearman’s correlation test were used.

**Results:** The highest vitreous VEGF concentration was in group B - 972.96 (743.33-1149.13) and was higher than in group A - 69.22 (37.33-225.15) and in group C - 19.93 (1.15-32.17) (p<0.001). There was a positive correlation between VEGF vitreous concentration and glucose level in group A patients (Rho=0.410; p=0.027).

**Conclusion:** As a treatment before PPV surgery, PRP showed to be effective in the reduction of VEGF levels, which also highlighted a decrease in complications during and postoperatively.

**Abbreviations:** DRS = Diabetic Retinopathy Study, PDR = proliferative diabetic retinopathy, VEGF = vascular endothelial growth factor, PRP = pan-retinal photocoagulation, PPV = pars plana vitrectomy, HbA1c = glycosylated hemoglobin, ETDRS = Early treatment diabetic retinopathy study, ESR = erythrocyte sedimentation rate, BCVA = best corrected visual acuity, OCT = optical coherent tomography, ILM = internal limiting membrane, PHACO = phacoemulsification, IOL = intraocular lens, ELISA = Enzyme-linked immunosorbent assay test, AUC = area under the curve, DME = diabetic macular oedema, TDR = tractional retinal detachment, VMT = vitreomacular traction

## Introduction

According to The Diabetic Retinopathy Study (DRS), half of the untreated eyes with proliferative diabetic retinopathy (PDR) result in severe vision-threatening sequelae [**1**], making PDR one of the major causes of preventable blindness in the world. The primary event in PDR is abnormal vasoproliferation caused by an excess production of mitogen, vascular endothelial growth factor (VEGF) [**2**], with VEGF-A as a key component with the strongest angiogenic stimulation [**3**]. Retinal hypoxia induces the process of angiogenesis, with VEGF as a main angiogenic factor [**4**] causing a stimulus for proliferation and migration of endothelial cells along with microvascular permeability and new vessel survival [**5**]. 

Pan-retinal photocoagulation (PRP) was the first and only treatment choice for PDR and is recently supplemented by intravitreal anti-VEGF therapy, increasing the treatment success rate. The main goal of PRP is to decrease retinal ischemic area, neovascularization, and vascular permeability and therefore decrease further VEGF production [**6**]. Pars plana vitrectomy (PPV) is a surgical treatment of PDR when other treatment options are either insufficient or unsuccessful. 

Diabetic retinopathy can be prevented by systemic control of blood pressure, blood glucose, and glycosylated hemoglobin (HbA1c) levels, which are all responsible for VEGF expression [**7**]. Many studies have already reported an increased level of VEGF in eyes with PDR [**1**,**2**,**8**]. Although various recent studies are describing VEGF levels before and after anti-VEGF therapy [**2**], there is a limiting number quantitatively comparing the effect of previously performed PRP on intravitreal VEGF concentration.

The aim of this study was to determine the intravitreal concentration of VEGF in eyes with PDR and to evaluate the effects of previous PRP on its level. 

## Methods

This is a cross-sectional study performed at the Clinical Centre University of Sarajevo (Clinic for Eye disease and Clinical immunology) during 2014 and 2016. It included 90 eyes, surgically treated with PPV after other treatment options were either insufficient or unsuccessful. Patients were divided into three groups: group A (30 patients) were PDR patients with previous PRP following Early treatment diabetic retinopathy study (ETDRS), group B (30 patients) were PDR patients without previous PRP, and Group C (30 patients) was a control group of patients who underwent PPV due to the indication unrelated to diabetes. 

Inclusion criteria were age (older than 18), signed informed consent, and PPV surgery. Exclusion criteria were the presence of eye disease non-related to DR, previously performed PPV or anti-VEGF therapy, and malignant or chronic inflammatory disease. The study was conducted under the principles of the Helsinki Declaration with a signed informed consent by all the patients. 

The data obtained were related to gender, age, insulin and disease duration, and another ophthalmological and systemic disease. A blood sample was taken as a part of the standard preoperative examination for general anaesthesia, including the number of erythrocytes, leukocytes, erythrocyte sedimentation rate (ESR), HbA1c, glucose level, urea, and creatinine. 

The ophthalmological examination included best-corrected visual acuity (BCVA) using the Snellen chart, intraocular pressure measurement using Goldman applanation tonometry, anterior and posterior segment examination on slit lamp, eye ultrasound, fundus photography, and optical coherent tomography (OCT). All the patients underwent PPV surgery. Moreover, all the patients underwent endolaser photocoagulation intraoperatively, but only 30 patients (group A) underwent PRP preoperatively. PRP was performed in several sessions following ETDRS protocol, using topical anaesthesia and mydriasis, with panfundoscope VOLK lens. Usually, 3000 laser burns, of a size of 300μ, and a pulse duration of 150ms, using a power that left visible whitish retinal marks (200-250mW), were employed. 

PPV was performed in local retrobulbar, or general endotracheal anaesthesia (ETA). After standard preoperative preparation of the surgical field, peritomy was performed 3.5-4.0 mm from the limbal area, depending on the lens status. Sclerotomies and trocar placement were performed above and below lateral rectus and above medial rectus muscle insertion (20G or 23G). Some eyes had a combined PPV and phacoemulsification (PHACO) performed with acrylic intraocular lens (IOL) implantation. The vitreous gel was removed, following removal of pathological vitreoretinal tissue, with or without internal limiting membrane (ILM) peeling and occasionally retinotomies in cases of combined proliferative vitreoretinopathy (PVR) and severe retinal contraction. Endolaser photocoagulation was performed in all eyes and intraocular tamponade used either air, expanding gasses (SF6 or C3F8), or silicon oil.

The vitreous sample was obtained during PPV in a sterile 1 ccm silicone tube, specifically designed for this study, and placed in an aspiration line to obtain 0.5-1.0 ccm undiluted vitreous, before the infusion system was opened. After a centrifuge, the sample was frozen at -80ºC. VEGF concentration was determined using an Enzyme-linked immunosorbent assay test (ELISA) in a Clinical immunology laboratory, using Qvantikine ELISA original set (USA R&D Systems, Inc. 614 McKinly Place, NE, Minneapolis, MN 55413). 

*Statistical analysis*

SPSS 21.0 was used for statistical analysis. The results were expressed as mean value, median, standard error of arithmetic mean, and interquartile range (25th–75th percentiles). The following tests were used: Shapiro-Wilk (for testing the significant difference in deviation from a normal distribution), Kruskal-Wallis nonparametric tests and Mann-Whitney U test (for variables that deviated from a normal distribution), and ANOVA (for normally distributed dependent variables). Since there was no normal distribution, a correlation was determined using Spearman’s test. For the determination of optimal cut-off values of VEGF in PDR with and without previous PRP receiver, operating characteristic curve (ROC curve) was used with the corresponding area under the curve (AUC). ROC curve accuracy rate was calculated with a 95% confidence interval (95% CI). P-value <0.05 was used as statistically significant. 

## Results

The difference between age, IOP, systolic and diastolic pressure between the groups, did not show any statistical significance (**Table 1**). The lowest BVCA was in group B 0.001 (0.001-0.01) and was significantly lower compared to both group A 0.02 (0.007-0.10) and group C 0.01 (0.001-0.325). Group A also had higher BVCA compared to the control group (p=0.03). There was a significant difference in BCVA between the groups (p<0.001; p<0.05) (**Table 1**). Duration of insulin therapy was significantly longer in group A compared to group B (p=0.007) (**Table 1**). Duration of diabetes was significantly longer in group B than in group A (p<0.001) (**Table 1**).

**Table 1:** Baseline characteristics

Variables	Group A (n=30)	Group B (n=30)	Group C (n=30)	P value
Age (years)	59.20±8.35	61.76±10.75	61.0±9.70	0.574
IOP (mmHg)	16 (14-18)	18 (15-18.25)	14 (12-18)	0.139
BCVA	0.02 (0.007-0.1)	0.001 (0.001-0.01)	0.01 (0.001-0.325)	<0.05
Systolic pressure (mmHg)	170 (140-180)	167.5 (155-180)	160 (130-180)	0.985
Diastolic pressure (mmHg)	90 (83.75-95)	90 (88.75-100)	87.5 (80-96.25)	0.091
Duration of diabetes (years)	19.5 (16-22.25)	27 (23.5-32.0)	-	<0.001
Duration of insulin therapy (years)	14 (11.75-16.25)	11 (6-17)	-	0.007
Data presented as mean ± standard deviation (X ± SD) and as median with 25-75 percentile interquartile range; Group A - PDR with previous PRP (n=30); Group B - PDR without previous PRP (n=30); Group C - control group (n=30), IOP - intraocular pressure; BCVA - best corrected visual acuity				

**Table 2** shows laboratory parameters in all three groups of patients. ESR, HbA1c, glucose, urea, and creatinine levels showed a significant difference between the examined groups (p<0.05). The number of erythrocytes and leukocytes did not show a significant difference (p>0.05). 

**Table 2:** Laboratory parameters

Variables	Group A (n=30)	Group B (n=30)	Group C (n=30)	P value
Erythrocyte number/ L	4.6 (4.1-4.87)	4.11 (3.8-4.6)	4.1 (3.35-5.76)	0.636
Leukocyte number/ L	6.6 (4.8-6.8)	6.6 (5.4-9.6)	5.8 (4.95-7.95)	0.221
ESR (mm)	11 (9-18)	18 (11-32.25)	16 (9.5-17.5)	0.005
HbA1C (%)	7 (6.6-7.7)	9.8 (8.85-11)	6.9 (6.45-7.05)	<0.001
Glucose (mmol/ L)	8.25±1.96	10.08±3.43	6.09±1.25	<0.001
Urea (µmol/ L)	6 (4.9-7.7)	7.1(6.3-8.95)	6,6 (5.3-8.45)	0.031
Creatinine (µmol/ L)	71 (64-99)	112 (73-141.5)	83 (58.5-269)	0.007
Data presented as mean ± standard deviation (X ± SD) and as median with 25-75 percentile interquartile range; Group A - PDR with previous PRP (n=30); Group B - PDR without previous PRP (n=30); Group C - control group (n=30), ESR - erythrocyte sedimentation rate				

**Fig. 1:**
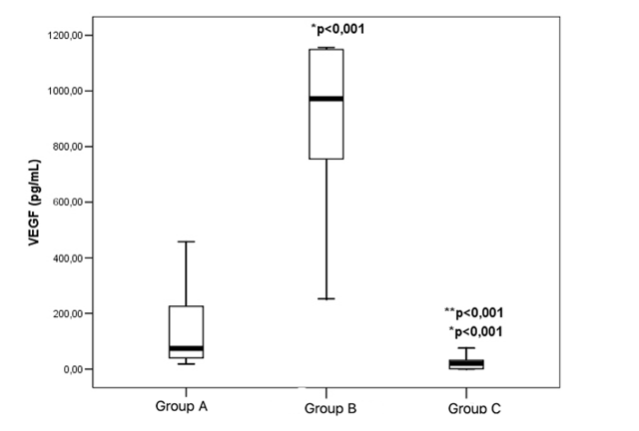
VEGF vitreous concentration Data presented as median with 25-75 percentile interquartile range; Group A - PDR with previous PRP (n=30); Group B - PDR without previous PRP (n=30); Group C - control group (n=30), *compared to group A; ** compared to group B; VEGF - vascular endothelial growth factor

The highest vitreous VEGF concentration was in the group without previously performed PRP (group B): 972.96 (743.33-1149.13) and was higher than the vitreous VEGF concentration in the group with previous PRP (group A): 69.22 (37.33-225.15) (p<0.001). It was also significantly higher compared to the controlled group, without angiogenic activity (group C): 19.93 (1.15-32.17) (p<0.001). A significant higher VEGF concentration was found in patients with previously performed PRP (group A) compared to the controlled group (group C) (p<0.001) (**Fig. 1**). 

**Table 3** shows the optimal cut-off value of VEGF determined by ROC curve 462.98 pg/ ml. The area under the curve (AUC) was 0.975 with a 95% CI of 0.975 (p=0.001). Maximal sensitivity was 83.30%, maximal specificity 100.00%, positive predictive value 100.00%, and negative predictive value 85.20%. 

**Table 3:** Sensitivity and specificity of vitreous VEGF as a differentiating biomarker of patients with PDR with and without previous PRP

Variable	Optimal cut off	AUC (95% CI)	Sensitivity (%)	Specificity (%)	Positive predictive value (%)	Negative predictive value (%)	P <
VEGF (pgl/ ml)	462.98	0.975	83.3	100	100	85.2	0.001
AUC - area under the curve							

**Fig. 2:**
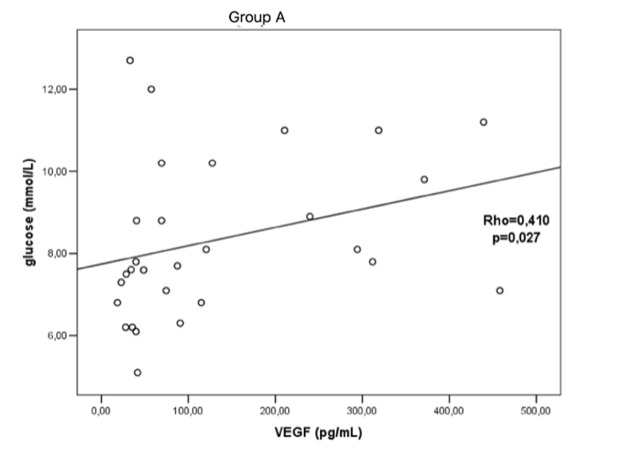
Correlation between VEGF vitreous concentration and blood glucose level in patients with previously performed PRP (group A) Rho – Spearman’s rank correlation coefficient VEGF - vascular endothelial growth factor

There was a positive significant correlation between VEGF vitreous concentration and glucose level in patients with PDR and previously performed PRP (Rho=0.410; p=0.027) (**Fig. 2**). There was no significant correlation in the other two groups between VEGF concentration and laboratory and baseline parameters. 

## Discussion

The findings of this study confirmed the hypothesis that the levels of vitreous VEGF are not only higher in PDR patients compared to the control group, but also higher in patients without PRP pre-treatment than in patients with PRP pre-treatment. 

VEGF has a central role in angiogenic processes and is responsible for endothelial proliferation and migration, increased vascular permeability, and new blood vessel survival [**5**]. Despite its vital importance, its hyperproduction can become a risk factor for the development of serious neovascularization and PDR progression [**9**]. VEGF incites endothelial cell growth and neovascularization along with the increase in vascular permeability in the ischaemic retina [**10**]. VEGF levels are increased in vitreous and aqueous humour as well as in excised hard macular exudates in patients with PDR and diabetic macular oedema (DME) [**11**]. Therefore, targeting VEGF is the main purpose of a treatment plan in PDR. 

On-time follow-ups and treatment of PDR using PRP are of high importance and were the golden standard and the only treatment option for PRP before the appearance of anti-VEGF therapy. Many studies have shown that the level of VEGF decreases after PRP [**9**,**11**,**12**]. Our study had preoperative PRP performed only in group A. The reasons for not performing PRP pre-treatment in group B included the inability of performance due to decreased transparency of optical media (cataract or haematovitreus), the presence of tractional retinal detachment (TRD) with macular involvement, severe fibrovascular proliferation, and the presence of preretinal or epiretinal membranes or other mechanical components that could only be resolved during PPV. Many other researches also confirm the importance of PRP pre-treatment [**13**,**14**]. Bakhritdinova et al. highly embrace preoperative PRP due to stabilization of proliferative processes, prevention of vitreomacular traction (VMT) formations, thus keeping the anatomical relations and visual acuity more stable [**14**]. PRP improves retinal function, influences faster and more successful recovery time and has a lower number of complications [**14**,**15**]. Some authors explain the decrease in VEGF levels by a decrease in angiogenic stimuli after PRP, which further prevents and reduces VEGF production, thus causing the regression of neovascularization [**15**]. Itaya et al. have reported an increased level of VEGF within one week of scatter photocoagulation [**16**]. Some studies report a significant reduction in aqueous humor VEGF after intravitreal bevacizumab and pre-treatment with PRP, which lasted up for 8 weeks [**17**]. They claim that the VEGF level increases highly on the first day, has its peak values on the third day, followed by a decrease from the third to the fifth day [**16**]. It can be concluded that lower VEGF levels increase the recovery effects, which speaks in favor of anti-VEGF therapy as the additional therapy for angiogenesis reduction [**18**].

Intravitreal anti-VEGF therapy reduces VEGF levels and presents a successful treatment for DME [**6**]. Even though there is an improvement in DME after anti-VEGF injection, ischemic retinal areas still produce VEGF and cause further neovascularization [**6**]. Takamura et al. showed that PRP prevents the increase in central retinal thickness after intravitreal bevacizumab injection [**6**]. Their study has also shown that PRP is useful in preventing the recurrence of DME even in wider areas of non-perfused retina [**6**]. This means that ischaemia and hypoxia are the reason for DME recurrence, which lead to the conclusion that a combination of PRP and anti-VEGF injections is important for successful PDR and DME. Several studies are reporting that anti-VEGF therapy is superior to PRP and causes better improvement in visual acuity [**19**], but PRP is still frequently used for non-responders and for non-reliable patients who skip follow-ups and monthly injections [**20**]. Anti-VEGF has also proven to be very successful preoperatively and intraoperatively due to significant decrease of intraoperative and postoperative haemorrhage [**21**]. It was also reported that VEGF concentration can be used as a predictive factor for certain postoperative complications, such as vitreous haemorrhage or glaucoma [**13**,**14**].

Wang et al. have reported that progressive forms of PDR have higher concentrations of VEGF compared to both less progressive disease and to control group [**13**]. Our results confirmed their conclusions as we have shown that PDR patients have significantly higher vitreous VEGF concentration compared to the control group. Our study showed high sensitivity (83.3%) and high specificity (100.0%) of VEGF as a differentiating biomarker in patients with PDR with and without PRP. Therefore, it can be concluded that the VEGF level can be a potential risk factor for PDR development and progression. 

There was a significant positive correlation between vitreous VEGF concentration and blood glucose levels in patients with previous PRP. This correlates with other studies in which patients with good diabetes control have lower serum and vitreous VEGF levels [**22**]. The same authors reported a significant positive correlation between vitreous and serum VEGF concentration, hence the conclusion that anti-VEGF therapy would be beneficial in PDR progression reduction. Even though there are many proofs that abnormal glucose levels lead to vascular complications, it is still impossible to state that regulation of blood glucose levels eliminates the risk for complications. Increased VEGF production in diabetic patients can be a result not only of periods of hyperglycaemia and hypoglycaemia, but also of exogenous insulin intake or genetic factors. Studies suggest that blood glucose level fluctuations cause damage known as cell pseudohypoxia, which leads to the synthesis of VEGF [**23**]. This study results could be improved with increased significance if an additional group of patients preoperatively treated with anti-VEGF was included. Further improvements of this study could have been made by longer follow ups and measuring these long-term results, and comparing them between groups. 

## Conclusion

The strength of this study is a relatively large sample size of surgically treated patients. The results of the study further prove the benefits of PRP and suggest that it is an important therapeutic tool in the improvement of the outcome of a challenging and severe diagnosis, such as PDR. 

Increased vitreous VEGF level in PDR patients suggests its role in the pathology of PDR. PRP as a treatment before PPV surgery has shown to be effective in the reduction of VEGF levels that also affects a decrease in complications during and postoperatively. 

**Conflicts of Interest statement**

The authors declare that there are no conflicts of interest. 

**Informed Consent and Human and Animal Rights statement**

Informed consent has been obtained from the patient included in this study. 

**Authorization for the use of human subject**

Ethical approval: The case report related to human use complies with all the relevant national regulations, institutional policies, is in accordance with the tenets of the Helsinki Declaration, and has been approved by the review board of the Veneto Eye Bank Foundation, Venice, Italy. 

**Acknowledgments**

None.

**Sources of Funding**

None.

**Financial Disclosure(s)**

None.
